# Quantitative assessment of diffuse myocardial fibrosis in II-type diabetes mellitus patients using T1 mapping technique: preliminary data

**DOI:** 10.1186/1532-429X-18-S1-O46

**Published:** 2016-01-27

**Authors:** Nicola Galea, Federica Ciolina, Marco Francone, Elisa Giannetta, Riccardo Pofi, Carlo Catalano, Iacopo Carbone

**Affiliations:** 1 grid.7841.aDepartment of Radiological, Oncological and Pathological Sciences, Sapienza - University of Rome, Rome, Italy; 2 grid.7841.aDepartment of Experimental Medicine, Sapienza University of Rome, Rome, Italy

## Background

In diabetic cardiomyopathy (DCM), ventricular remodelling consists in a progressive impairment of myocardial contraction (evolving from diastolic to combined diastolic-systolic dysfunction) occurring regardless of ischemic heart disease, hypertension or other macrovascular complications, which ultimately leads to heart failure. Early stages of DCM are asymptomatic and characterised by initial contractile disfunction and various degree of myocardial fibrosis, that may not be recognised by traditional cardiology tests. Our purpose was to detect myocardial fibrotic infiltration in DM-II patients by using T1-mapping technique with extracellular volume fraction (ECV) measurement.

## Methods

Forty diabetic patients (31 men;age 64.5 yrs) with preserved ventricular function and no history of ischemic cardiopathy and 20 controls underwent to CMR. Imaging protocol included: modified Look-Locker sequence before and 20 minutes after 0.2 mmol/kg gadoterate meglumine injection; T2 mapping; ventricular function module; conventional T2-weighted and late gadolinium enhanced (LGE) imaging. Global native T1 (nT1), T2 and ECV values were calculated and correlated to clinical features such as symptoms, glycated haemoglobin (HbA1c), duration of disease and therapy. Pearson Correlation, Mann-Whitney test and unpaired T-test were used for statistical analysis.

## Results

Patient group had higher nT1 and ECV values compared to controls (1046 ± 54 ms vs. 975 ± 38 ms, 28.9 ± 3.7%vs.24.8 ± 4.3% respectively, p < 0.05 for both), whereas no significant differences occurred in T2 measurements (45.8 ± 2.4 ms vs. 47.0 ± 2.8 ms respectively, p: 0.23).

nT1 and ECV showed direct correlations with HbA1c (nT1:r2 = 0.98, p < .0001; ECV:r2 = 0.95, p < .0001) and disease duration (nT1:r2 = 0,98 p < .0001; ECV:r2 = 0.55, p < .0001) in diabetic patients. An inverse correlation was found between T2 and HbA1c (r = -0,46; p = 0,009).

Areas of ischemic LGE were found in two patients as marker of silent infarction.

## Conclusions

Diabetic patients with preserved ventricular function have increased nT1 and ECV as reflection of silent slight inflammation and diffuse myocardial fibrosis, which degree correlates with HbA1c and disease duration.

